# Vitamin B5 Reduces Bacterial Growth *via* Regulating Innate Immunity and Adaptive Immunity in Mice Infected with *Mycobacterium tuberculosis*

**DOI:** 10.3389/fimmu.2018.00365

**Published:** 2018-02-26

**Authors:** Wenting He, Shengfeng Hu, Xialin Du, Qian Wen, Xiao-Ping Zhong, Xinying Zhou, Chaoying Zhou, Wenjing Xiong, Yuchi Gao, Shimeng Zhang, Ruining Wang, Jiahui Yang, Li Ma

**Affiliations:** ^1^School of Laboratory Medicine and Biotechnology, Institute of Molecular Immunology, Southern Medical University, Guangzhou, China; ^2^Division of Allergy and Immunology, Department of Pediatrics, Duke University Medical Center, Durham, NC, United States

**Keywords:** tuberculosis, vitamin B5, pro-inflammation, antibacteria, macrophages, immune cells

## Abstract

The mechanisms by which vitamins regulate immunity and their effect as an adjuvant treatment for tuberculosis have gradually become very important research topics. Studies have found that vitamin B5 (VB5) can promote epithelial cells to express inflammatory cytokines. We aimed to examine the proinflammatory and antibacterial effect of VB5 in macrophages infected with *Mycobacterium tuberculosis* (MTB) strain H37Rv and the therapeutic potential of VB5 *in vivo* with tuberculosis. We investigated the activation of inflammatory signal molecules (NF-κB, AKT, JNK, ERK, and p38), the expression of two primary inflammatory cytokines (tumor necrosis factor and interleukin-6) and the bacterial burdens in H37Rv-infected macrophages stimulated with VB5 to explore the effect of VB5 on the inflammatory and antibacterial responses of macrophages. We further treated the H37Rv-infected mice with VB5 to explore VB5’s promotion of the clearance of H37Rv in the lungs and the effect of VB5 on regulating the percentage of inflammatory cells. Our data showed that VB5 enhanced the phagocytosis and inflammatory response in macrophages infected with H37Rv. Oral administration of VB5 decreased the number of colony-forming units of H37Rv in lungs of mice at 1, 2, and 4 weeks after infection. In addition, VB5 regulated the percentage of macrophages and promoted CD4^+^ T cells to express interferon-γ and interleukin-17; however, it had no effect on the percentage of polymorphonuclear neutrophils, CD4^+^ and CD8^+^ T cells. In conclusion, VB5 significantly inhibits the growth of MTB by regulating innate immunity and adaptive immunity.

## Introduction

Tuberculosis, caused by *Mycobacterium tuberculosis* (MTB), has been recognized as a major infectious disease worldwide (1). In 2015, the World Health Organization estimated that there were 10.4 million new cases of active tuberculosis and 1.4 million deaths worldwide (2). The people with tuberculosis have generally weight loss and micronutrient deficiencies because of change metabolic processes, decreasing appetite and causing a reduction in food intake (3). Furthermore, the people with active tuberculosis have the poorer nutritional status than people with latent tuberculosis throughout the world (4). However, micronutrients are the key factors of cell-mediated immunity, which is the key host defense against tuberculosis. Micronutrient deficiencies will lead to a depressed immune response in the body, which increases the susceptibility to active tuberculosis and delays recovery (5–7).

An increasing number of researchers have begun to explore the mechanisms by which vitamins regulate immunity and their effects as an adjuvant treatment for tuberculosis. Recently, vitamin (V) A, C, and D have received the most attention, and the mechanisms by which they regulate immunity have already been elucidated. VA plays a vital role in both T- and B-lymphocyte function, the generation of antibody responses, and macrophage activity (8, 9). Vitamin C increases reactive oxygen species levels and DNA damage (10). VD is a key component of autophagy and cytokine production in macrophages and regulates lipid metabolism in MTB infection (11). Thus, we aimed to investigate the vitamins that could help the body build an effective immune response to resist infection by MTB and improve tuberculosis treatment outcomes.

After MTB infection, phagocytic cells including macrophages and recruited neutrophils will be recruited to the lung and form the early host responses (12, 13). Neutrophils and macrophages further facilitate the activation of antigen-specific CD4^+^ and CD8^+^ T cells to promote an anti-MTB adaptive immune response (14). As the first line of immune defense against MTB, macrophages provide a major habitat for MTB to remain dormant in the host for years. Toll-like receptors expressed on macrophages can recognize pathogen-associated-molecular patterns on MTB and mediate the activations of multiple inflammatory signaling pathways to initiate a tailored immune response toward the invading pathogen. Furthermore, productions of proinflammatory cytokines, such as tumor necrosis factor (TNF) and interleukin-6 (IL-6) (15), play a crucial role in pathogen clearance (16). Some studies reported that vitamin B5 (VB5) (pantothenic acid) can promote the expression of inflammatory cytokines in epithelial cells (17, 18). Thus, we hypothesized that VB5 could possibly enhance the inflammatory response of macrophages and further help the body to resist the MTB infection.

In this study, we found that VB5 promoted the phagocytosis by macrophages and was helpful to limit growth of intracellular mycobacteria in macrophages *in vitro*. Furthermore, *in vivo* VB5 was beneficial for the body to defend against the mycobacterial infection by promoting the maturity of macrophages and increasing differentiation of Th1 and Th17 cells in mice with MTB infection without excessive inflammation.

## Materials and Methods

### Animals

Specific pathogen-free C57BL/6J mice, 6 weeks old, were purchased from the Experimental Animal Center of Southern Medical University.

All animal experiments in this study were carried out in accordance with the recommendations in the Guide for the Care and Use of Laboratory Animals of the National Institutes of Health. All experimental protocols were reviewed and approved by the Medical Ethics Board and the Biosafety Management Committee of Southern Medical University (approval number SMU-L2016003).

### Infection of Mice and Oral Administration of Vitamin B1

The MTB H37Rv (ATCC 27294, the same below) infection of mice was performed described as previously described (19). Every mouse was infected ~200 bacteria to the lung. To confirm the dose of MTB H37Rv inoculation, the bacterial titers in the lungs of at least two mice were determined at 24 h (h) after infection. Followed, VB5 (MP Biomedicals, USA), isoniazid (INH), and phosphate buffer solution (PBS) were alternatively administered orally. The VB5 solution (7.3 g/l) was prepared by dissolving 220 mg VB5 in 30 ml PBS. The INH solution was prepared as a positive control. PBS was as a negative control. Oral administration was started from the day after infection (day 1) and performed every day until the end of 4 weeks. For VB1-treated mice, the VB5 dose (200 μl/mouse) was equivalent to 10 µg VB1/100 g body weight. For control mice, INH (2 g/l) or PBS was given in the same manner as that for VB5-treated mice.

### Spleen and Lung Weights and Colony-Forming Unit

Spleens and lungs were removed from mice at the indicated time points. Weights of the spleens and lungs were measured used an electronic scales. Bacterial burden was determined by plating serial dilutions of spleen and lung homogenates onto 7H10 agar plates (BD Biosciences, USA) supplemented with 10% OADC. Plates were incubated at 37°C in 5% CO_2_ for 3–4 weeks before counting colonies. All infections were performed in triplicate.

### Fluorescence-Activated Cell Sorting (FACS) Analysis

The following anti-mouse antibodies were from eBioscience: FITC-anti-F4/80, APC-anti-CD80, PE-Cy7-anti-CD86, PE-anti-MHC-II, PE-anti-CD11b, FITC-anti-Gr-1, APC-Cy7-anti-CD3, PerCP-Cyanine5.5-anti-CD4, APC-anti-CD8a, PE-anti-IFN-γ, and FITC-anti-IL-17. For intracellular cytokine staining, cells were stimulated with PMA (50 ng/ml, Enzo Life Sciences) and ionomycin (1 nM, Enzo Life Sciences) in the presence of brefeldin A (1 mg/ml, Enzo Life Sciences) for 4–5 h. Surface staining was performed in FACS buffer (0.5% BSA, 2 mM EDTA, 0.02% sodium azide in PBS) in the presence of Fc receptor blocking antibody (BioLegend) for 20 min at 4°C. Cells were fixed in 4% paraformaldehyde (Electron Microscopy Sciences) for 20 min at room temperature followed by permeabilization (0.1% BSA, 0.5% saponin in PBS) or with the BD Cytofix/Cytoperm Fixation and Permeabilization Kit. Cytokine staining was performed in permeabilization buffer for 20 min at 4°C. Data acquisition and analysis were performed using BD LSRFortessa X-20 (BD Bioscience) and FlowJo software (Tree Star, Ashland, OR, USA) respectively.

### Culture of Bone Marrow-Derived Macrophages (BMDMs), Mycobacterial Infection, and Stimulation of VB5

Bone marrow cells were taken from C57BL/6J mice and placed on cell culture dishes (96 mm × 22 mm; CELLTER, China) at 37°C/5% CO_2_ in DMEM (Corning, NY, USA) containing 10% fetal bovine serum (FBS; Corning, NY, USA). The cells differentiated into macrophages induced by granulocyte macrophage colony stimulating factor (GM-CSF; 100 ng/ml; PeproTech, USA) until the seventh day. BMDMs were placed on a 12-well cell culture plates (CELLTER) for 48 h at 37°C/5% CO_2_ in DMEM containing 10% FBS. Then, cells were persistently infected with MTB H37Rv until the indicated time. VB5 (10 μM) was added every 24 h.

### *In Vitro* MTB Killing Assay

Bone marrow-derived macrophages were infected with MTB H37Rv at a multiplicity of infection (MOI) of 5 at 37°C with 5% CO2. Then, cells were extensively washed with prewarmed PBS to remove non-adherent bacteria. The cells were incubated at 37°C with 5% CO2 for indicated time, and then were lysed in 1 ml of distilled water. Bacterial burden was determined by plating serial dilutions of cell lysates onto 7H10 agar plates supplemented with 10% OADC. Plates were incubated at 37°C in 5% CO_2_ for 3–4 weeks before counting colonies. All infections were performed in triplicate.

### Western Blotting

Cells were lysed in lysis buffer containing PhosSTOP phosphatase inhibitor Cocktail (Roche Diagnostics, Germany) and 1 mM DTT for different time periods. The cell debris was pelleted by centrifugation at 12,000 × *g* at 4°C, and 1 µl of each supernatant was used for determination of protein concentration using a bradford protein assay (Bio-Rad, USA). Cell lysates were separated by SDS-PAGE and then transferred to PDFV membranes using a wet transfer cell. After blocking with TBST containing 5% bovine serum albumin (BSA; VETEC, China) for 1 h, the membranes were incubated with the primary antibodies specific for phospho-p65, total p65, phospho-AKT, total AKT, phospho-JNK, total JNK, phospho-ERK, total ERK, phospho-p38, total p38, and GAPDH (1:1,000; Cell Signaling Technology, USA) in TBST-5% BSA at 4°C overnight. The membranes were then washed three times with TBST for a total of 30 min, followed by incubation with antibodies goat anti-rabbit (1:1,000) or anti-mouse IgG-horseradish peroxidase conjugates (1:2,000; Cell Signaling Technology) for 1 h at room temperature and three washes with TBST for a total of 30 min. The immunoblots were visualized by enhanced chemiluminescence (ECL; Thermo Fisher Scientific, USA).

### Real-time PCR

RNA was extracted from BMDM cells with the TRIzol Reagent according to the manufacturer’s protocol. The cDNA was synthesized with TransScript One-Step gDNA Removal and cDNA Synthesis SuperMix (TransGen Biotech, China) with oligo-dT primer according to the manufacturer’s protocols. The expression of mouse genes encoding TNF-α, IL-6, and β-actin was assessed by real-time PCR with TransStart Top Green qPCR SuperMix (TransGen Biotech) on a Mastercycler ep realplex4 (Eppendorf, Germany). The PCR conditions included an initial step at 94°C for 30 s, followed by 45 cycles of amplification and quantification (94°C for 5 s, 60°C for 30 s). β-Actin was used as an internal control. Relative gene expression levels were calculated using the 2^−ΔΔCt^ method (20). The forward primer and reverse primer for mTNF-α were 5′-CACAGAAAGCATGATCCGCGAC-3′ and 5′-TGCCACAAGCAGGAATGAGAAGAG-3′. The forward primer and reverse primer for mIL-6 were 5′-GTCCGGAGAGGAGACTTCAC-3′ and 5′-CTGCAAGTGCATCATCGTTGT-3′. The forward primer and reverse primer for mβ-Actin were 5′-GATTACTGCTCTGGCTCCTAGC-3′ and 5′-GACTCATCGTACTCCTGCTTGC-3′.

### Enzyme-Linked Immunosorbent Assay (ELISA)

Cell culture supernatants were collected and assayed for cytokines. Cytokine production was measured by ELISA of mouse TNF-α and IL-6 (ExCell Bio, China) according to the manufacturer’s protocol.

### Statistics

All experiments were performed at least twice. Unpaired two-tailed *t*-tests or one-way ANOVA were used to determine the statistical significance of differences between data groups with Prism 5 (Prism; Graphpad Software, Inc.). A *P* value of <0.05 was considered significant.

## Results

### VB5 Was Helpful to Limit Growth of Intracellular Mycobacteria in Macrophages

**Figure 1:**
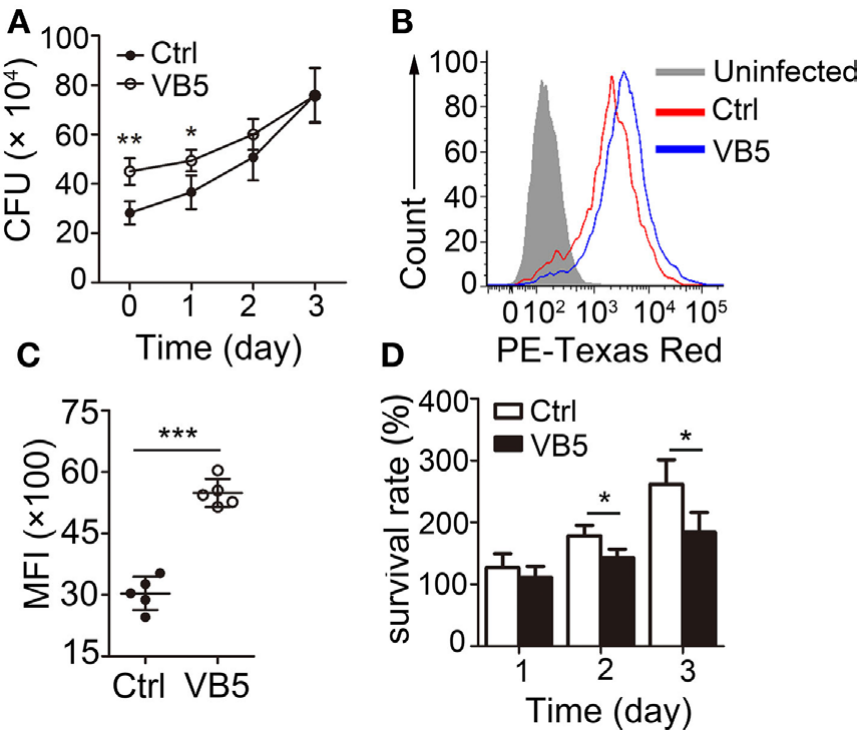
Vitamin B5 (VB5) promoted the *Mycobacterium tuberculosis* (MTB) phagocytosis by macrophages and the clearance of intracellular mycobacteria in macrophages. **(A)** Bone marrow-derived macrophages (BMDMs) were pretreated with VB5 followed by MTB H37Rv infection, and intracellular viable bacteria were detected with colony-forming unit (CFU) assays at 0, 1, 2, and 3 day postinfection. **(B,C)** BMDMs were pretreated with phosphate buffer solution or VB5 (10 µM) for 24 h and then challenged with Texas-Red-labeled MTB H37Rv (multiplicity of infection = 5) for 1 h. **(B)** Phagocytosis of MTB H37Rv was determined by flow cytometry. **(C)** Mean fluorescence intensity (MFI) was calculated. **(D)** Survival rate was calculated *via* the method that the numbers of viable bacillus at different times divided the number of viable bacillus at 0 day. Data shown are the mean ± SD. *P < 0.05, **P < 0.01. Data are representative of three independent experiments with similar results.

Macrophages are the major cells that provide the habitat for MTB and the first and main cells that kill and eliminate MTB. First, we wanted to determine whether VB5 could play a role directly in the clearance of intracellular mycobacteria. We found that there was a remarkable increase in the number of bacilli ingested by VB5-treated BMDMs compared to the control BMDMs in the initial stage of infections (Figure 1A). However, there was a weaker trend in the growth of intracellular viable bacilli in VB5-treated BMDMs than those in the control group at 1, 2, and 3 days postinfection (Figure 1A). To further assess whether VB5 affect macrophage-mediated phagocytosis of MTB, we pretreated BMDMs with VB5, followed by fluorescent Texas-Red-labeled MTB H37Rv infection, and phagocytosis was analyzed by flow cytometry. Our results showed that VB5 had major effects on MTB phagocytosis by macrophages (Figures 1B,C). Furthermore, the percentage of viable mycobacteria showed decreased mycobacterial survival in VB5-treated cells compared to baseline (Figure 1D). Cell viability was measured after infection with MTB H37Rv for the indicated times by propidium iodide staining after pretreatment with dexamethasone (Figure S1 in Supplementary Material). Our results showed no major decrease in cell viability of either vehicle or VB5 treatment. Collectively, these results implied that on one hand VB5 promoted phagocytosis by macrophages and that, on the other hand, VB5 was helpful to limit growth of intracellular mycobacteria in macrophages.

### VB5 Promoted the Innate Immune Response in Macrophages after Mycobacterial Infection

**Figure 2:**
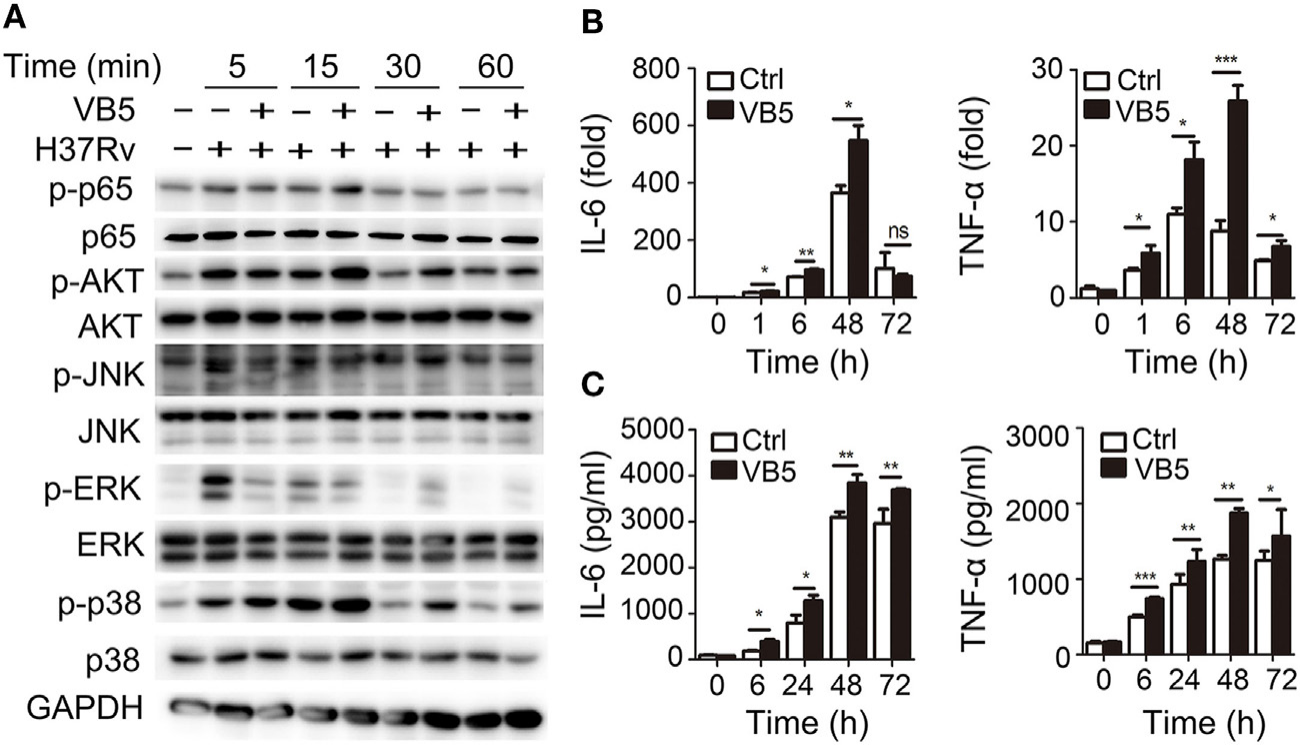
Vitamin B5 (VB5) promoted the innate immune response in macrophages after mycobacterial infection. Bone marrow-derived macrophages were pretreated with VB5 followed by *Mycobacterium tuberculosis* H37Rv infection. **(A)** Western blot analysis of the phosphorylation status of NF-κB, AKT, JNK, ERK, and P38. GAPDH is as an internal control. These results are from a representative experiment (*n* = 3). **(B)** Tumor necrosis factor (TNF)-α and interleukin-6 (IL-6) mRNA expression determined by real-time PCR. **(C)** IL-6 and IL-23 secretion for indicated time points was measured by enzyme-linked immunosorbent assay. *P < 0.05, **P < 0.01, ***P < 0.001. Data are representative of three independent experiments with similar results.

Vitamin B5 was helpful in the clearance of intracellular mycobacteria in macrophages. Thus, we sought to explore the effect of VB5 on innate immunity during mycobacterial infection. First, we examined the major inflammatory signal molecules to explore the effect of VB5 on inflammatory signaling pathways of macrophages infected with H37Rv. We found that VB5 promoted the phosphorylation of NF-κB, AKT, and p38. With respect to ERK, VB5 suppressed the phosphorylation early; however, as time progressed, the effect turned into that of promotion (Figure 2A; Figure S2 in Supplementary Material). Because TNF-α and IL-6 play a role in pathogen clearance by macrophages, we analyzed the mRNA and protein expression of these inflammatory cytokines. We found that H37Rv-induced mRNA levels of TNF-α and IL-6 were substantially increased in VB5-treated BMDMs (Figure 2B). Similarly, protein levels of TNF-α and IL-6 were substantially increased in the VB5-treated BMDMs (Figure 2C). However, there was no difference in protein levels of IL-4, IL-10, and IL-13 between VB5-treated BMDMs and control group (Figure S3 in Supplementary Material). Overall, these results demonstrated that VB5 mainly had a proinflammatory effect in macrophages after mycobacterial infection.

### VB5 Promoted the Maturity of Macrophages *In Vitro*

**Figure 3:**
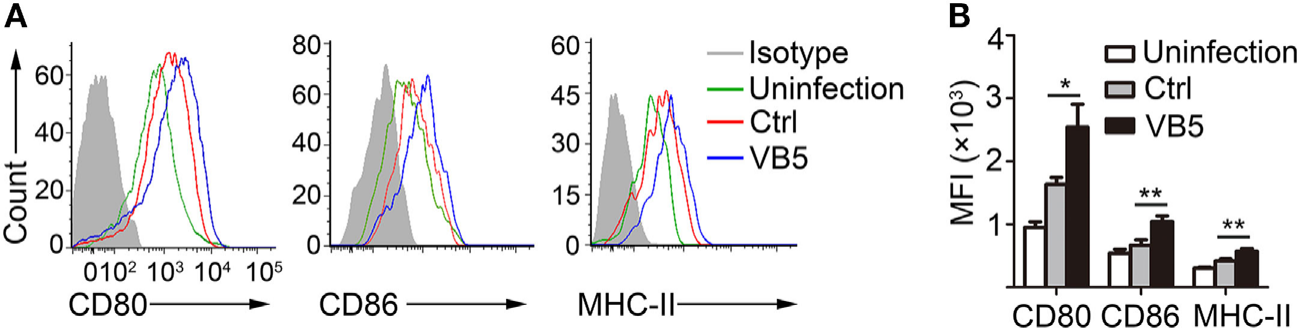
The expressions of CD80, CD86, and MHC-II on bone marrow-derived macrophages (BMDMs). **(A)** BMDMs were pretreated with vitamin B5 (VB5) or not (Ctrl) for 24 h and infected with *Mycobacterium tuberculosis* H37Rv for 24 h. The expressions of CD80, CD86, and MHC-II were detected *via* flow cytometry. Untreated and uninfected BMDMs were as the negative controls (Uninfection). Data are representative of three independent experiments with similar results. **(B)** The expressions of CD80, CD86, and MHC-II were assessed as mean fluorescence intensity (MFI).

The previous results showed that VB5 promoted phagocytosis by macrophages (Figure 1A), which indicated that VB5-treated macrophages had the stronger potential activation status. Therefore, we investigated the expression levels of activation biomarkers, CD80, CD86, and MHC-II in BMDMs. The results showed that VB5-treated macrophages displayed higher levels of CD80, CD86, and MHC-II expression than the control group (Figures 3A,B). These data indicated that VB5 promoted the maturity of macrophages *in vitro*.

### VB5 Led to Decreased Mycobacterial Growth in Mice

**Figure 4:**
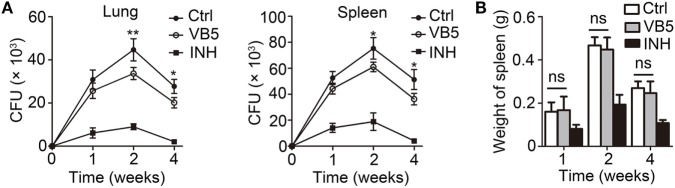
The anti-bacillus effect of vitamin B5 (VB5) in mice with *Mycobacterium tuberculosis* infection. C57BL/6J mice were infected with H37Rv (~200 bacteria/mouse). Oral administration with phosphate buffer solution, VB5, and INH was started from the day after infection (day 1) and continued for 1, 2, and 4 weeks alternatively. The lungs and spleens were analyzed at indicated time. **(A)** Colony-forming units (CFUs) were obtained from the lungs and spleen cell lysates by serial dilution and plating on 7H10 agar in triplicate. The colonies were counted after 4 weeks. **(B)** Spleen weights were detected. Data shown are the mean ± SD. *P < 0.05, ***P < 0.001. Data are representative of three independent experiments with similar results.

Vitamin B5 was helpful in the clearance of intracellular mycobacteria in macrophages. We further sought to investigate whether VB5 could help the body to resist the infection by MTB. We examined the H37Rv burden in the lungs and spleens of infected mice after oral administration of VB5 or isoniazid or PBS (control group) for 1, 2, and 4 weeks. We found that MTB growth was significantly suppressed in the lungs and spleens of VB5- or INH-treated mice at all indicated time points (Figure 4A). In addition, we measured the spleen and lung weight to explore whether VB5 had a proinflammatory effect. The results showed that the spleen and lung weights of VB5-treated mice were similar to that of the control group (Figure 4B; Figure S4A in Supplementary Material). These results suggested that VB5 was beneficial to the body to defend itself against the mycobacterial infection without causing excessive inflammation.

### VB5 Decreased the Infiltration of Macrophages but Promoted the Maturity of Macrophages in Mice with MTB Infection

**Figure 5:**
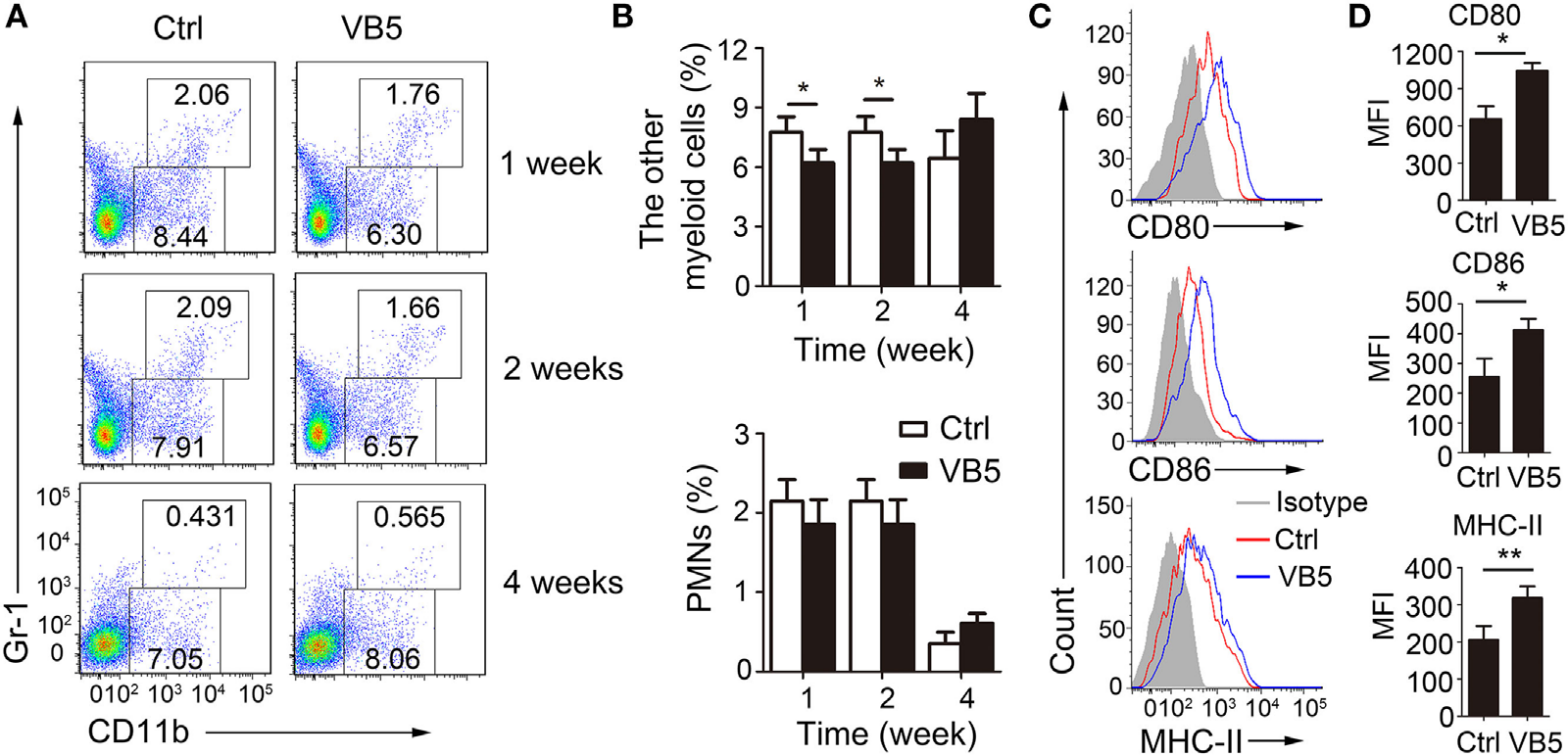
The effect of vitamin B5 (VB5) on the percentage of myeloid cells in lungs. Lung cells from H37Rv-infected mice treated with VB5 or untreated were harvested at 1, 2, and 4 weeks after infection. Flow cytometric analysis of lung cells stained with anti-mouse CD11b and anti-mouse Gr-1 antibodies. **(A)** The percentage of myeloid cells is displayed as dot plots. **(B)** The percentages of other myeloid cells and polymorphonuclear leukocytes (PMNs) in lungs were shown. **(C)** The expressions of CD80, CD86, and MHC-II were detected *via* flow cytometry. **(D)** The expressions of CD80, CD86, and MHC-II were assessed as mean fluorescence intensity (MFI). Data shown are the mean ± SD. *P < 0.05. Data are representative of three independent experiments with similar results.

Next, we determined the percentage of immune cells in lungs of infected mice treated with VB5 or PBS by FACS analysis to explore how VB5 helps the body to build an effective immune response to resist the infection by MTB. Monocyte-macrophages and polymorphonuclear leukocytes (PMN) are the first cells that kill and eliminate MTB. First, we determined the percentages of monocyte-macrophages and neutrophils. The results showed that VB5 had no effect on the percentage of PMNs in lungs (Figures 5A,B). However, it decreased the other myeloid cells, which mainly included monocyte-macrophages at 1 and 2 weeks after infection (Figures 5A,B). In addition, there was no difference between the numbers of infiltrated cells in lungs of VB5-treated mice and control group (Figure S4B in Supplementary Material). Our previous results suggested that VB5 promoted the maturity of macrophages *in vitro*, thus, we investigated this effect *in vivo*. Similarly, we determined that increased expression levels of CD80, CD86, and MHC-II were seen on macrophages from VB5-treated mice compared with those obtained from untreated mice (Figures 5C,D). These results suggested that VB5 decreased the infiltration of macrophages, but promoted the maturity of macrophages in mice with MTB infection.

### VB5 Increased Differentiation of Th1 and Th17 Cells in Mice with MTB Infection

**Figure 6:**
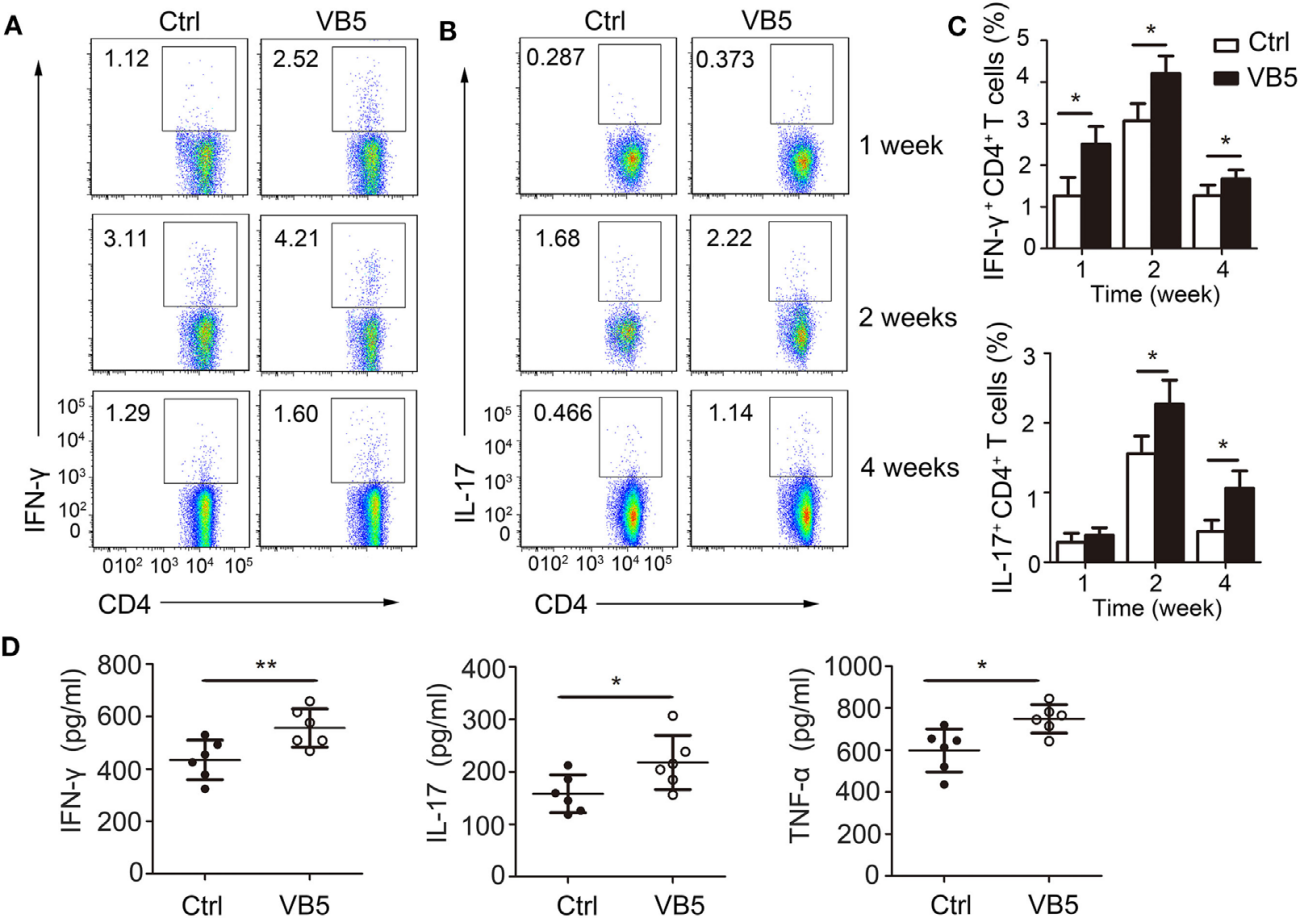
The effect of vitamin B5 (VB5) on intracellular IFN-γ and IL-17 in CD4^+^ T cells of lungs. Lung cells from H37Rv-infected mice treated with VB5 or untreated were harvested at 1, 2, and 4 weeks after infection. Flow cytometric analysis of lung cells stained with anti-mouse CD4, anti-mouse IFN-γ, and anti-mouse IL-17 antibodies. **(A)** The percentage of CD4^+^ T cells producing intracellular IFN-γ was displayed as dot plots. **(B)** The percentage of CD4^+^ T cells producing intracellular IL-17 was displayed as dot plots. **(C)** The percentages of CD4^+^ T cells producing intracellular IFN-γ and IL-17 were shown. **(D)** Concentration of IFN-γ, IL-17, and tumor necrosis factor (TNF)-α in the serums of mice with *Mycobacterium tuberculosis* infection. Data shown are the mean ± SD. *P < 0.05, **P < 0.01. Data are representative of three independent experiments with similar results.

Adaptive immune responses also play a vital role against MTB infection. Thus, we explored the effect of VB5 in the adaptive immune responses in mice with MTB infection. First, we found that VB5 had no effect on the percentage of CD4^+^ and CD8^+^ T cells in lungs (Figures S5A,B in Supplementary Material). As already recognized, Th1 and Th17 cells can recruit monocytes and granulocytes to infected sites and promote their antimicrobial activities by secreting inflammatory cytokines. We found that the percentages of IFN-γ and IL-17 were higher in the lungs from VB5-treated mice compared with those of control mice 1, 2, and 4 weeks after MTB infection (Figures 6A–C). However, the VB5 had no significant effect on the differentiations of Th2 in mice with MTB infection (Figure S5C in Supplementary Material). Furthermore, we determined that the serum from VB5-treated mice had increased concentrations of IFN-γ, IL-17 and TNF-α compared to serum from control mice (Figure 6D). The concentrations of anti-inflammatory cytokines IL-4, IL-10, and IL-13 had no significant differences between VB5-treated groups and control groups (Figure S5D in Supplementary Material). In addition, VB5 had no effect on the percentage of CD4^+^ and CD8^+^ T cells in lungs draining lymph node (Figure S6 in Supplementary Material). Together, these observations indicated that VB5 increased differentiation of Th1 and Th17 cells in mice with MTB infection.

## Discussion

Vitamins are vital as an adjuvant treatment for tuberculosis. However, not all immunoregulatory mechanisms of vitamins are clear (21). VA and VD have received the most attention and their regulatory mechanisms have been largely elucidated (22).

Here, we investigated the role of VB5 in the host immune response against mycobacterial infection. VB5 is a water-soluble B-complex vitamin. Previous reports have focused on the metabolic regulation of VB5 in glucose, fatty acids, and amino acids (23). Our results showed that VB5 could promote the phagocytosis by macrophages and was helpful in limiting growth of intracellular mycobacteria in macrophages. Furthermore, VB5 could regulate the maturity of macrophages and differentiation of Th1 and Th17 cells in mice. These findings provide a novel insight of VB5 in a regulatory mechanisms role.

A previous study suggested that VB5 is essential for maintaining keratinocyte proliferation and differentiation (24). Consistent with their study, our results showed that VB5 could regulate the maturity of macrophages. This suggests that VB5 may play a vital role in cell development. This is a possible mechanism related to the defense against tuberculosis.

Activation of proinflammatory pathways, including NF-κB, PI3K-AKT, p38, JNK, and ERK initiates the production of cytokines in innate immune cells such as macrophages, neutrophils, and dendritic cells (25). We found that VB5 can activate various inflammatory signal molecules (NF-κB, AKT, ERK, and p38) and can promote the expression of TNF-α and IL-6. These molecules function as anti-tuberculosis immune factors (13, 26). It was suggested that VB5 could affect innate immune response to limit the growth of MTB in macrophages. VB5 is involved in the synthesis of coenzyme A (CoA), which has long been recognized as an essential cofactor in biochemical reactions in various organisms (27). Previous studies showed that CoA plays a crucial role in the inflammatory process (23). Thus, VB5 may regulate the innate immune response through regulating the CoA level. Further study will be required to clarify this possibility.

Next, we utilized the mouse model of MTB infection to study the anti-MTB effect of VB5 *in vivo* and found that VB5 could decrease the growth of MTB. PMN and macrophages contribute to early defense against MTB (28). After assessing the percentage of PMN and macrophages in lungs using flow cytometry, we found that VB5 decreased the percentage of macrophages in lungs at 1 and 2 weeks after infection and elevated it at 4 weeks. In addition, VB5 had no impact on the percentage of PMN at each time point. Studies have found that apoptosis caused neutrophils to secrete anti-inflammatory cytokines to reduce the recruitment or activation of macrophages; however, necrosis is to the contrary (29). We hypothesized that VB5 might promote neutrophils to secrete anti-inflammatory cytokines to inhibit the recruitment or activation of macrophages in early infection, but stimulate neutrophils to secrete pro-inflammatory cytokines to promote the recruitment or activation of macrophages in late infection.

After activation, CD4^+^ T cells produce IFN-γ and IL-17 to enhance the ability of macrophages and PMNs to eliminate MTB (30). We found that VB5 could increase the expressions of IFN-γ and IL-17 in CD4^+^ T cells during MTB infection. Differentiation of macrophages mirrors and affects polarization of T cells *in vivo* (31, 32). *In vitro*, VB5 showed the ability that promoted the maturity of macrophages, thus, we considered that VB5 may regulate the polarization of T cells *in vivo via* functioning macrophages. It is thought that VB5 stimulates immune cells to secrete cytokines and that its functions are multiple.

Taken together, these findings suggest that orally administered VB5 significantly inhibits the *in vivo* growth of MTB *via* the regulation of innate immunity and adaptive immunity. Thus, orally administered VB5 may potentially have important therapeutic implications in the clinical management of tuberculosis.

## Ethics Statement

All animal experiments in this study were carried out in accordance with the recommendations in the Guide for the Care and Use of Laboratory Animals of the National Institutes of Health. All experimental protocols were reviewed and approved by the Medical Ethics Board and the Biosafety Management Committee of Southern Medical University (approval number SMU-L2016003).

## Author Contributions

SH, WH, and LM designed research; SH, WH, XD, WX, YG, SZ, RW, and JY conducted research; SH, WH, X-PZ, XZ, CZ, QW, and LM analyzed data; QW provided essential reagents or provided essential materials; SH, WH, and LM wrote the paper. LM had primary responsibility for final content. All authors read and approved the final manuscript.

## Conflict of Interest Statement

The authors declare that the research was conducted in the absence of any commercial or financial relationships that could be construed as a potential conflict of interest.
