# Supplementary material for: Vitamin B5 Reduces Bacterial Growth via Regulating Innate Immunity and Adaptive Immunity in Mice Infected with Mycobacterium tuberculosis
Source: Front Immunol. 2018 Feb 26;9:365. doi: 10.3389/fimmu.2018.00365 (PMC5834509; doi:10.3389/fimmu.2018.00365)
Supplement: Supplementary file 1 [file data_sheet_1.docx]

**Supplement figure 1**

**
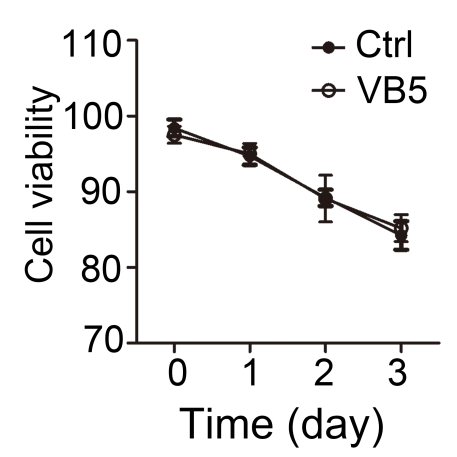
**

**Figure S1** Effects of VB5 on cell viability after mycobacterial infection. BMDM were pretreated with VB5 for 24 hr and then challenged with MTB H37Rv (MOI=5) for 1 hr. The infected cells were washed extensively with PBS to remove extracellular mycobacteria. Cells were collected after incubated for the indicated time and stained with propidium iodide (PI) to test cell viability. The percentage of cells viability was shown. Data shown are the mean ±SD. Data are representative of three independent experiments with similar results.

**Supplement figure 2**


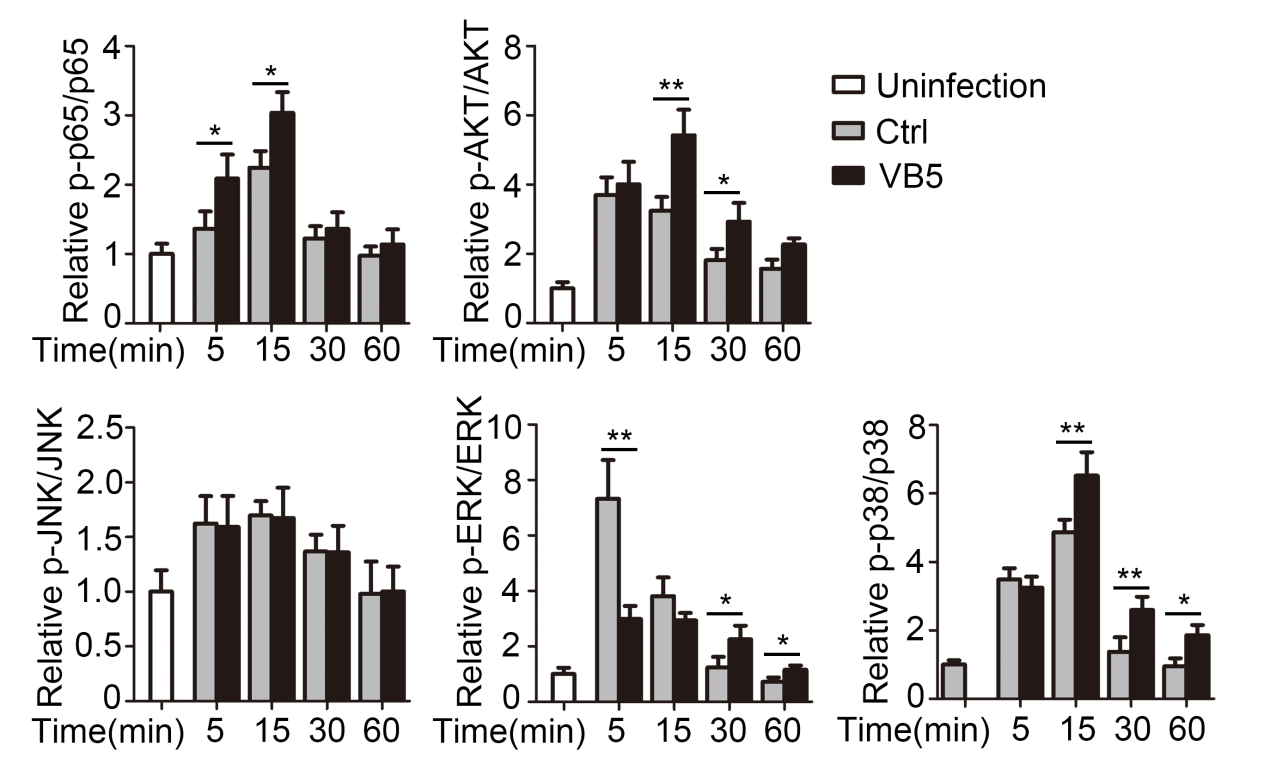


**Figure S2 Densitometry quantification of band intensity of Figure 2.** BMDMs were pretreated with VB5 followed by MTB H37Rv infection for appointed time. Western blot analysis of the phosphorylation status of NF-κB, AKT, JNK, ERK and p38. **P* < 0.05, ***P* < 0.01. Data shown are the mean ±SD. Data are representative of three independent experiments with similar results.

**Supplement figure 3**

**
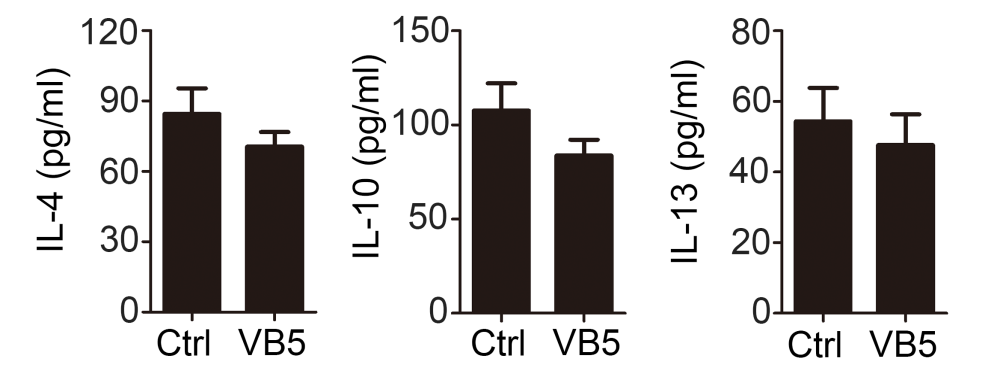
**

**Figure S3** **The Effect of VB5 on IL-4, IL-10 and IL-13**. BMDMs were pretreated with VB5 followed by MTB H37Rv infection. IL-4, IL-10 and IL-13 secretion for indicated time points was measured by ELISA. Data are representative of three independent experiments with similar results.

**Supplement figure 4**


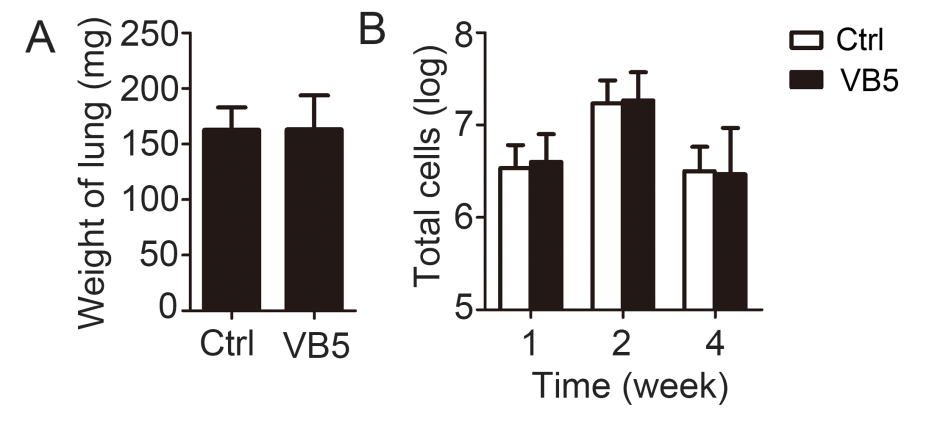


**Figure S4 Weight of lung and number of inflammatory cells in lungs of mice at 2 week after MTB infection.** C57BL/6J mice were infected with H37Rv (~200 bacteria / mouse). Oral administration with PBS or VB5 was started from the day after infection. **(A)** Lung weights were detected. **(B)** Lung cells from H37Rv-infected mice treated with VB5 or untreated were harvested at 2 weeks infection. Data shown are the mean ±SD. Data are representative of three independent experiments with similar results.

**Supplement figure 5**


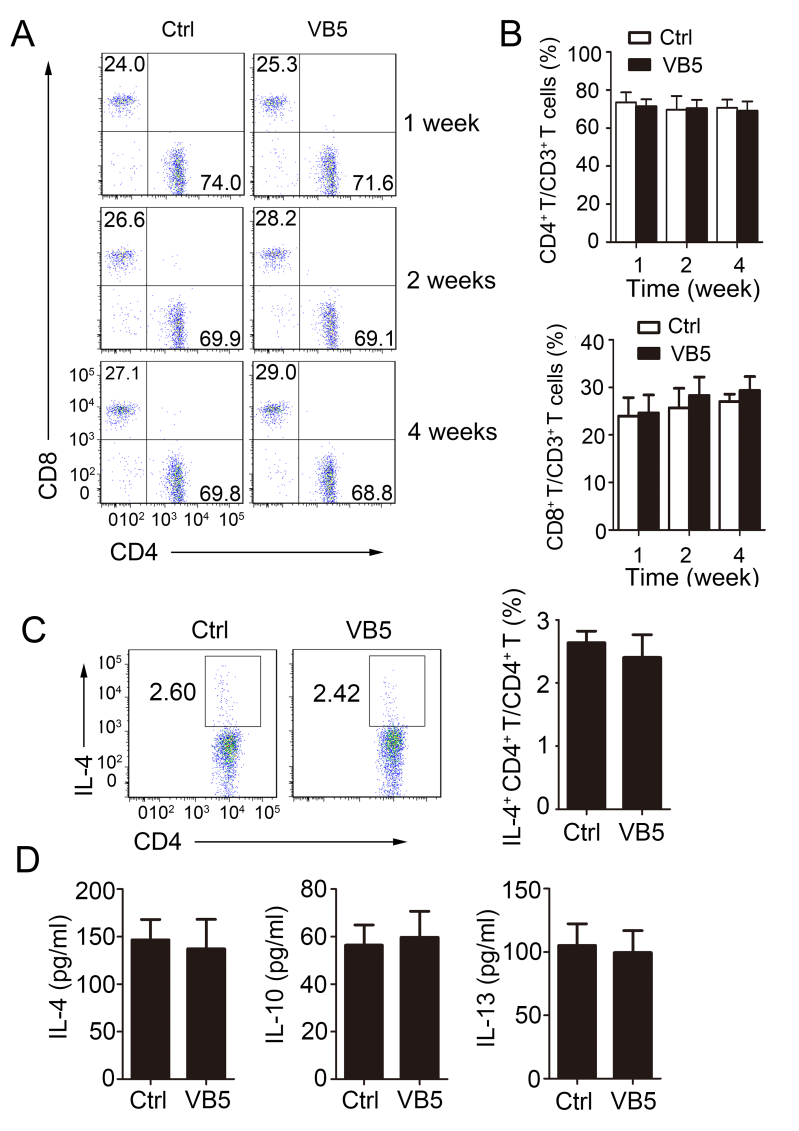


**Figure S5 The Effect of VB5 on the percentage of CD4^+^ and CD8^+^ T cells and anti-inflammatory cytokines.** Lung cells from H37Rv-infected mice treated with VB5 or untreated were harvested at 1 week, 2 weeks and 4 weeks after infection. Flow cytometric analysis of lung cells stained with anti-mouse CD4 and anti-mouse CD8 antibodies. **(A)** The percentage of CD4^+^ and CD8^+^ T cells in CD3^+^ T cells of lungs is displayed as dot plots. **(B)** The percentages of CD4^+^ T and CD8^+^ T cells in CD3^+^ T cells of lungs were shown. **(C)** The percentage of CD4^+^ T cells producing intracellular IL-4 was displayed as dot plots. (D) Concentration of IL-4, IL-10 and IL-13 in the serums of mice with MTB infection. Data shown are the mean ±SD. Data are representative of three independent experiments with similar results.

**Supplement figure 6**


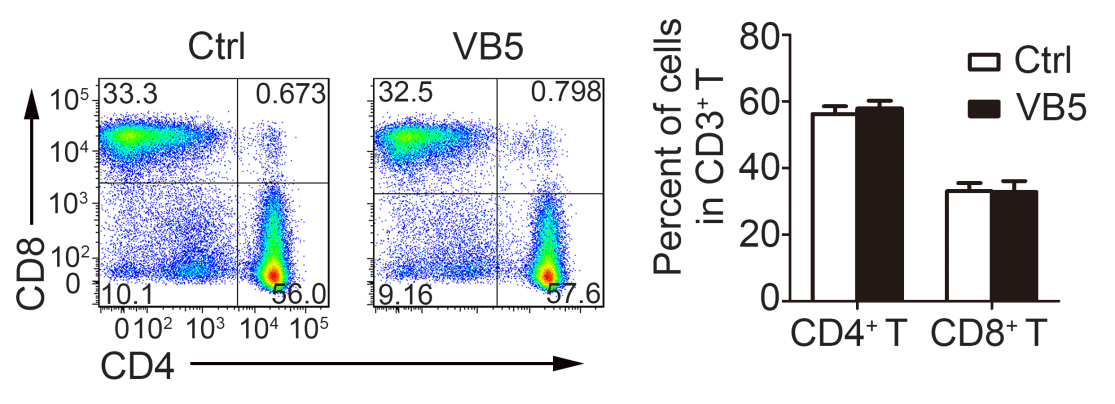


**Figure S6 The Effect of VB5 on the percentage of CD4^+^ and CD8^+^ T cells in lung draining lymph node.** Lung draining lymph node cells from H37Rv-infected mice treated with VB5 or untreated were harvested 2 weeks after infection. Flow cytometric analysis of lymph node cells stained with anti-mouse CD4 and anti-mouse CD8 antibodies. Data shown are the mean ±SD. Data are representative of three independent experiments with similar results.
